# Molecular Markers in Cutaneous Squamous Cell Carcinoma

**DOI:** 10.1155/2011/231475

**Published:** 2011-08-09

**Authors:** Anthony P. Tufaro, Jim C.-M. Chuang, Nijaguna Prasad, Alice Chuang, Tony C. Chuang, Anne C. Fischer

**Affiliations:** ^1^Department of Plastic & Reconstructive Surgery, Johns Hopkins Medical Institutions, Baltimore, MD, USA; ^2^Department of Oncology, Johns Hopkins Medical Institutions, Baltimore, MD, USA; ^3^Department of Surgery, Johns Hopkins Medical Institutions, Baltimore, MD, USA; ^4^Division of Gastroenterology, Department of Medicine, University of Chicago, Chicago, IL, USA; ^5^Division of Otolaryngology, Head and Neck Surgery, Department of Surgery, Samaritan Medical Center, Watertown, NY, USA; ^6^Department of Surgery and Cancer Immunobiology Center, UT Southwestern Medical, Dallas, TX, USA

## Abstract

Nonmelanoma skin carcinoma (NMSC) is the most frequent cancer in the USA with over 1.3 million new diagnoses a year; however due to an underappreciation of its associated mortality and growing incidence and its ability to be highly aggressive, the molecular mechanism is not well delineated. Whereas the molecular profiles of melanoma have been well characterized, those for cutaneous squamous cell carcinoma (cSCC) have trailed behind. This importance of the new staging paradigm is linked to the ability currently to better clinically cluster similar biologic behavior in order to risk-stratify lesions and patients. In this paper we discuss the trends in NMSC and the etiologies for the subset of NMSC with the most mortality, cutaneous SCC, as well as where the field stands in the discovery of a molecular profile. The molecular markers are highlighted to demonstrate the recent advances in cSCC.

## 1. Introduction

Nonmelanoma skin carcinoma (NMSC) is the most frequent cancer in the USA and worldwide [1]; it has been increasing in overall incidence since the 1960's at a rate of 3–8% per year [2]. With over 1.3 million new diagnoses of NMSC a year in the United States [3], it is both the diversity of types, of which there are 82, and biologic variability in phenotype that make the analysis of NMSC even more challenging. Although the incidence of basal cell carcinoma (BCC) exceeds cSCC by a 5 : 1 ratio, cSCC is associated with the burden of mortality with a disease-specific yearly mortality rate of 1% per year as reported in the early 1990's [4]. Despite the fact that the majority of these tumors present at early stages, cSCC accounts for the majority of NMSC deaths [5] and 20% of all skin-cancer-related deaths [6]. 

Multiple etiologies exist for cSCC, including environmental, genetic, viral and altered host immunity and virally mediated. The high incidence of cSCC and BCC is caused by the mutagenic effects of ultraviolet (UV) light which is intensified by geographic latitude [1, 7]. cSCC and BCC are more common in fair skinned and anatomic sites exposed to the sun, such as head, neck, and extremities: head and neck is the most common site. Other known risk factors are male sex, advanced age, immunosuppression (induced or acquired), human papilloma viruses (HPV), chronic inflammation, and genetic diseases manifested in the skin [7–9]. Immunocompromised states are associated with a marked escalation of cSCC of up to 64–250 times greater than that in the general population compared to the 10-fold increased risk in BCC, causing a reversal of the typical ratio in immunocompetent individuals from 5 : 1 to a range between 1 : 1.8 and 1 : 15 [10, 11]. Immunosuppression significantly impacts the biology of cSCC. In solid organ transplant patients, cSCC tumors tend to be numerous, exhibit a strong propensity to recur, and metastasize at a high rate regardless of lesional size [12]. Malignant lesions develop within 10 years after organ transplantation and up to 80% of these lesions contain HPV DNA [13]. HPV has also been associated with cSCC with evidence of a higher viral load of HPV DNA in immunosuppressed patients [13, 14]. Likewise cSCC that arises in sites of chronic inflammation, such as scars, sinus tracts, and burns, can also demonstrate more aggressive clinical behavior and a greater propensity to metastasize with an overall metastatic rate of 40% [6, 15]. Cutaneous genetically inherited skin conditions that have a known propensity of risk for developing cSCC are albinism, xeroderma pigmentosum, and epidermodysplasia verruciformis [5, 16, 17]. 

## 2. Staging

With a cure rate of greater than 90% for the routine lesion, and the large number of low risk lesions, the significance of the diverse spectrum and numerous subtypes of cSCC has been underappreciated given the often quoted 5-year recurrence and metastatic rates of 8% and 5%, respectively [6, 18, 19]. However recent changes to the American Joint Committee on Cancer Staging (AJCC) presented in the 7th Edition focus on identifying clinical parameters that portend a worse prognosis to identify and stage appropriately that subset of cSCC that progresses to metastatic disease [9]. The high risk characteristics determined by the 7th Edition AJCC Staging Manual include lesional size (>2 cm), and high risk features such as a depth of invasion (>2 mm, ≥Clark level IV), perineural invasion, tumor grade (poorly differentiated or undifferentiated), as well as high-risk anatomic sites (see Table 1). The paradigm shift in the 7th Edition recognizes that the classification of lesions with similar histopathologic features is more likely to have similar aggressive behavior and the features chosen were based on that behavior. For instance, tumor grade alone is significantly associated with mortality given a 5-year cure after therapy of 61.5% for poorly differentiated cSCC compared to 94.6% for well differentiated cSCC [6]. High risk histologic features were defined as showing poor differentiation, spindle cell characteristics, necrosis, high mitotic activity, and deep invasion [9]. Both the depth of invasion and presence of perineural invasion significantly correlate with prognosis: it clearly has been shown that thicker lesions have a higher rate of nodal metastases and recurrence. In fact a depth of >4 mm thickness or ≥Clark Level IV is associated with a twofold increased rate of recurrence or 5-fold increase metastatic rate [6]. Similarly, perineural invasion is associated with a 5-fold increase in both the recurrence rate and metastatic rate [20]. Although not identified in the 7th Edition, other histologic features that are important in prognosis include lymphovascular invasion and the presence of inflammatory features such as the presence of eosinophils and plasma cells [21]. cSCC in immunocompromised patients or those that arise in scars, sinus tracts, or burns all demonstrate a more aggressive biologic phenotype with a greater metastatic rate of up to 40% [6, 15, 22]. So subsets with a worse prognosis are critical to correctly stage and classify together in order to appropriately reveal a previously unrecognized metastatic potential. Likewise recurrent or persistent disease portends a worse survival of 78% 5-year survival compared to 97% for a primary lesion [20, 23]. Since the incidence of metastatic cSCC is low, the outcome of metastatic cSCC is not universally recognized as dismal at a-5 year survival of less than 35% [6]. 

Prognostic features have not been universally accepted in cSCC and the histologic features delineated above are advised for uniformity in standardizing clinical staging guidelines. Since cSCC is typically deemed to have a good prognosis, the variants with the worst biology, by not being grouped together, are underpowered numerically in an outcome analysis. Thus the variety of subtypes of cSCC certainly necessitates a comprehensive clinicopathologic classification system to group variants of SCC based on their biologic aggressiveness or indolence. The 7th Edition introduces the histologic features that need to be tracked and classified; yet a clinicopathologic classification system would allow lesions to be stratified based on biologic behavior and thus patients accordingly to optimize therapy. Clinicopathologic classification of cSCC has been characterized by Cassarino et al. [24] to stratify lesions based on their malignant potential (Table 2). In the era of personalized medicine, molecular markers have been used in many tumors to prognosticate and risk-stratify patients. Given the relative lack of recognition of the growing incidence of cSCC and the inability to track the worst subset of cSCC given the abundance of low risk lesions and the practice of not banking or staging lesions, these molecular studies have been relatively limited compared to the field of melanoma. A summary of the current literature on the molecular markers in cSCC highlights some promising areas of research.

## 3. Pathogenesis

### 3.1. Viral Pathogenesis

The increased incidence of cSCC in immunocompromised patients compared to BCC suggests a mechanism of viral pathogenesis. HPV is important in immunosuppressed patients, which are known to have higher levels of HPV DNA in cSCC lesions. Evidence of a higher viral load has been reported in cSCC in organ transplant patients with up to 80% of lesions containing HPV DNA [14]. However the variable quantity of HPV in immunocompetent individuals can range between 27–70% depending on detection techniques [13, 17]. Thus the type of HPV, *β*-papillomavirus species 2, is more often associated with cSCC as opposed to the total amount of HPV DNA present [17]. Three theories have been suggested for the mechanism of HPV carcinogenesis: (1) UV radiation-induced immunosuppression to explain enhanced interaction between HPV and UV radiation [25, 26], (2) E6/E7 oncoprotein-related changes in p53 and Rb tumor suppressor gene, and (3) integration of HPV DNA disrupting genomic stability [17, 25]. Viral expression of E6 and E7 oncoproteins can inactivate p53 and Rb tumor suppressor genes, leading to an uncontrolled system of cell proliferation and apoptosis [27].

Association of viral pathogens such as human papillomavirus (HPV) with head and neck squamous cell cancer (HNSCC), especially oropharyngeal cancer has been recognized over the past two decades. HPV16 is the most common genotype in these tumours, whereas HPV6 and HPV11 can also be found in a minority of these cancers, implying that these low-risk HPV types are not entirely benign in HNSCC. HPV DNA is closely associated with poorly differentiated cancers, positive lymph nodes, and late-stage disease, which portend a worse diagnosis. HPV status is also associated with p16 expression and HPV+ tumours are less likely to harbour p53 mutations [28]. A subset of HNSCC patients who had HPV 16 infection confers a better prognosis. On the other hand, *β* papillomaviruses (*β*-HPVs) also play a role in the tumorigenesis of cSCC as shown by both European and US studies [29]. However, no high-risk types have been identified although there is an association of *β* species 1 in SCC. Other viruses, such as polyomavirus (MCPyV) have been shown to be causative agent in Merkel cell carcinoma [30]. 

### 3.2. Allelic Imbalance and Loss of Heterozygosity

The genetic progression model for head and neck squamous cell carcinoma (HNSCC) demonstrates that loss of heterozygosity (LOH) is common during the progression from premalignant lesion to malignant tumors [31]. Tumor suppressor genes (TSGs) are usually found in the area of loss rendering the cells more susceptible to tumorigenesis [32].

Several regions of chromosomal loss are identified in HNSCC. One of the most common regions, 9p21, has been reported in both HNSCC [33] and cSCC [34]. This region contains several TSGs, including p16INK4A (CDKN2A), p15INK4B, and MTAP. Allelic imbalances are also found in other regions of cSCC, including LOH on 3p, 2q, 8p, and 13 and allelic gain on 3q and 8q [35]. Such studies indicate that allelic imbalance and LOH are recognized and relevant events in cSCC and can be used for early diagnosis and tumor surveillance.

### 3.3. Epigenetics

Epigenetics refers to the molecular mechanisms that regulate gene expression without changes in the DNA sequence. Epigenetic alterations include DNA methylation and histone modifications, which consist of methylation, acetylation, phosphorylation, ubiquitination, and sumoylation, chromatin remodeling and microRNAs [36–38]. Changes in genomic DNA methylation associated with cancer include global DNA hypomethylation and gene-specific hyper- or hypomethylation. Tumor progression involved chromatin-mediated changes such as DNA methylation yet the role of histone variants in tumorogenesis is unclear. All of these modifications of gene expression have been associated with the development of various tumor types, including HNSCC and cSCC [39, 40]. A higher frequency of FOXE1 promoter hypermethylation was found in cSCC compared to normal skin, indicating that FOXE1 may be a target for aberrant methylation in cSCC [39].

Epigenetic dysregulation is thought to be involved in tumor biology and progression and thus may be relevant biomarkers for clinical prognosis [39]. Promoter DNA methylation gene panels have been described for screening of primary HNSCC [41, 42], for determination of tumor recurrence and assessment of margin status during surgery. Recent evidence does show that the loss of histone variants, such as macro-H2A, positively correlates with an increasing malignant phenotype of melanoma cells and that macro-H2A is a critical component of chromatin that suppresses malignant progression of melanoma [43]. However, a determination of methylation gene panels relevant in cSCC is yet to be established. To date there has been no correlation of the significance of global histone modifications to prognosis in cutaneous SCC although studies have shown them to be applicable in esophageal SCC [44]. A combination of different genes from different pathways may allow for a better determination of the aggressiveness of cSCC to better correlate to prognosis.

### 3.4. RNA and microRNA

Messenger RNA (mRNA) and microRNA (MiRNA) profiles have been described in both HNSCC and cSCC [45]. Several mRNA biomarkers for cSCC were identified, including CCR10, CCL27, MUC4, p16, MMP2, and MMP9 [46]. MicroRNAs play a role in regulation of mRNA. A recent study has demonstrated that a distinct microRNA profile is modulated by UV radiation [47]. 

### 3.5. Mitochondrial Mutation

Mitochondrial mutation in HNSCC has been well reported; however, only a few studies show the association of mitochondrial DNA mutation and cSCC [48]. Several regions of mitochondrial DNA were reported, including displacement loop (D-loop) [49] and other regions [50]. Therefore, mitochondrial mutations may correlate in the future with the phenotypic behavior of cSCC.

## 4. Conclusions

The molecular mechanisms that underlie the development of cutaneous skin cancers are poorly understood. Even the spectrum of biologic behavior has been slow to be characterized given the previously very generic clinical criteria used to distinguish low risk lesions from a more aggressive lesions. Recent changes in the classification of the staging paradigm has better captured the histologic features of this more aggressive subset to allow for a more precision in identifying the worst subset. Incorporating a clear clinicopathologic classification system into stratifying lesions is necessary to better cluster them by their biology besides just histologic features.Thus molecular analysis can potentially profile that subset with biomarkers chosen to best correlate with the biologic phenotype.

## Figures and Tables

**Table 1:** High risk factors for NMSC tumor characteristics*.

*Histologic differentiation*	
Poor differentiation	
Spindle cell characteristics	
Necrosis	
High mitotic activity	
Deep invasion	
*Depth of invasion *	
>2 mm	
Clark level ^3^IV	
*Perineural invasion*	
*High risk anatomic sites*	
Nonglabrous Lip	
Ear	
*Advanced T stage (T3 and T4)*	
Bony extension or involvement	
Maxilla, mandible, orbit, temporal bone	
Perineural invasion	
Invasion of skull base	
Invasion of axial or appendicular skeleton	

*7th Edition of American Joint Commission on Cancer Staging Manual [18].

**Table 2:** Clinicopathologic classification for cSCC*.

*High risk behavior cSCC*	
Bowen's disease with invasion	
Adenosquamous carcinoma	
cSCC in proliferating pilar tumor/cysts	
Tricholemmal cysts	
Epidermoid cysts	
Desmoplastic cSCC	
De novo cSCC	
cSCC in chronic conditions	
Ulcers, sinus tracts, burns	
Osteomyelitis	
Chronic inflammatory disorders	
Radiation-induced cSCC	
*Indeterminate risk cSCC*	
Clear cell SCC	
Signet ring cell	
Papillary cSCC	
Pigmented cSCC	
Follicular cSCC	
cSCC from benign adnexal cyst	
*Putative cSCC versus low grade cSCC*	
Keratoacanthoma (KA)	
Not a bona fide cSCC	
Low grade behavior	
Giant KA,	
Subungual KA	
KA with Immunosuppression	

*Modified Classification system from Cassarino et al. [24].
